# Multiple Bragg reflection by a thick mosaic crystal. II. Simplified transport equation solved on a grid

**DOI:** 10.1107/S2053273320002065

**Published:** 2020-04-16

**Authors:** Folkmar Bornemann, Yun Yvonna Li, Joachim Wuttke

**Affiliations:** a Zentrum Mathematik M3, Technische Universität München, 85747 Garching, Germany; b Jülich Centre for Neutron Science at MLZ, Forschungszentrum Jülich GmbH, 85747 Garching, Germany

**Keywords:** mosaic crystals, multiple scattering, Darwin–Hamilton equations, spectral collocation

## Abstract

The generalized Darwin–Hamilton equations [Wuttke (2014 ▸). *Acta Cryst*. A**70**, 429–440] describe multiple Bragg reflection from a thick, ideally imperfect crystal. These equations are simplified by making full use of energy conservation, and it is demonstrated that the conventional two-ray Darwin–Hamilton equations are obtained as a first-order approximation. Then an efficient numeric solution method is presented, based on a transfer matrix for discretized directional distribution functions and on spectral collocation in the depth coordinate. Example solutions illustrate the orientational spread of multiply reflected rays and the distortion of rocking curves, especially if the detector only covers a finite solid angle.

## Abstract 2

To describe multiple Bragg reflection from a thick, ideally imperfect crystal, the transport equations are reformulated in three-dimensional phase space and solved by spectral collocation in the depth coordinate. Example solutions illustrate the orientational spread of multiply reflected rays and the distortion of rocking curves, especially for finite detectors.

## 1. Introduction   

In a preceding paper, designated as Part I (Wuttke, 2014*a* ▸), multiple Bragg reflection from a thick, ideally imperfect crystal was studied mainly by analytical means. The planar two-ray transport equations of Darwin (1922 ▸) and Hamilton (1957 ▸) were generalized to account for out-of-plane trajectories. Expanding these equations into a recursive scheme led to some asymptotic results, but did not provide a practicable solution algorithm for the generic case with crystals of finite thickness. Reflection probabilities depend strongly on propagation directions, and with each reflection the next reflection probability can vary by orders of magnitude. This makes the transport equations ill conditioned, and straightforward Monte Carlo simulations inefficient and unreliable.

In this paper, a completely different solution method is presented. Instead of following individual rays through forward and backward reflections, we study reflection-order-independent fluxes (current distributions) *I* as a function of propagation direction 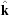 and penetration depth *z*. They are governed by a system of linear ordinary differential equations in *z* with separated boundary conditions [equations (1) and (4) below]. We present *spectral collocation* as a practicable solution method. Solutions are iterated with increasing numbers of collocation points until a required accuracy is reached. Our algorithm is fast enough to be used interactively or/and within complex instrument simulations.

This paper can be read independently of Part I. We re-de­ri­ve most of the theory, making it simpler and more generic. By consequential use of energy conservation, we get rid of one phase-space dimension. By positing translational invariance along the surface of the mosaic plate, two other dimensions are eliminated. This reduction to three argument dimensions is the precondition for an efficient numeric solution on a grid.

Large parts of the theory are now formulated coordinate free. Block normals 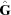 that fulfill the Bragg condition are parameterized by a polar instead of a Cartesian coordinate; this eliminates an apparent singularity that forced us in Part I to exclude near-backscattering from consideration. Furthermore, the transport equations are simplified and generalized by removal of any reference to two distinct beams.

While all worked-out examples assume a simple geometry with the mean crystallite normal collinear to the mosaic normal, our formalism can be used with any other orientational distribution. One application we have in mind is beam deflection by a rotating stack of tilted mosaic crystals of highly oriented pyrolytic graphite as used in the phase-space transform chopper of third-generation neutron backscattering spectrometers (Meyer *et al.*, 2003 ▸; Frick *et al.*, 2006 ▸; Wuttke *et al.*, 2012 ▸).

In Section 2, we derive the mathematical model to be studied. Discrete 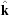 grids are chosen in Section 3. In Section 4 our numeric solution method is presented and verified against the two-ray model. Example solutions are shown in Section 5 and conclusions drawn in Section 6. Some derivations, computational details and special cases can be found in Appendices *A* [App appa] [App appb] [App appc]–*D* [App appd]. The supporting information provides the source code and additional documentation of the software *MultiBragg* developed along with this work.

## 2. The mathematical model   

### 2.1. Crystal model and current distribution   

Following Darwin (1922 ▸), a mosaic crystal is modeled as an assembly of perfectly crystalline blocks that are to some degree orientationally disordered. In an *ideally imperfect crystal* every block is so thin that it reflects at most a small fraction of the incident beam. Primary extinction and multiple reflections within a block can be neglected. As in Part I, we consider a *thick*, ideally imperfect crystal, consisting of so many block layers that secondary extinction and multiple reflections are of practical importance.

Since reflections from different blocks add incoherently, the adequate description level is classical transport theory. Our task is to compute the stationary *flux* (current distribution) 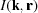. Only *elastic* diffraction shall be fully accounted for. Inelastic scattering will be dealt with by a loss term. Accordingly, the wavenumber *k* is a conserved quantity, and can therefore be dropped from the argument list of 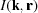, leaving over a dependence on the propagation direction 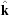.

**Figure 1:**
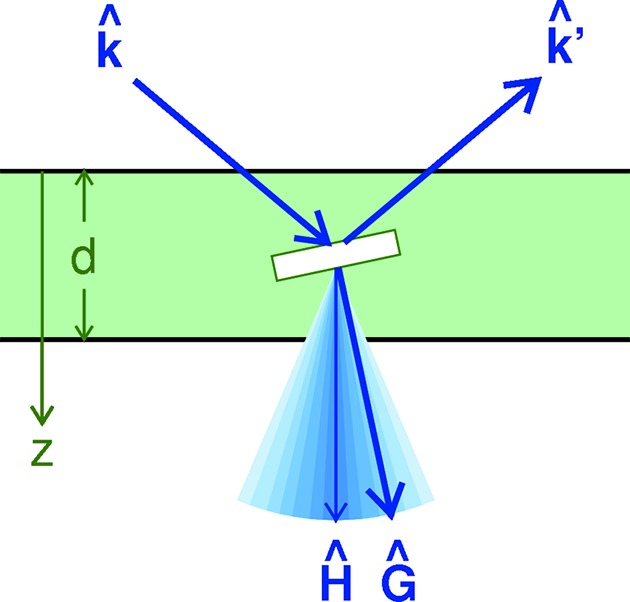
Bragg reflection by a crystalline block within a mosaic plate. Block normals 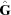 are distributed around 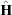, as indicated by the blue cone. The angular width of the distribution is grossly exaggerated; typically, it is a few degrees only. Here, and in all specific examples in this work, we have chosen 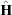 to be collinear with the real-space depth direction 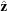. Much of our theory also holds for 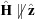.

As in Part I, we concentrate on a mosaic crystal in the form of a *slab* that can be approximated as an *infinite plate* (Fig. 1 ▸). Altogether the flux is projected from six-dimensional phase space to the three-dimensional function 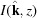. This opens the possibility of solving the boundary problem with manageable effort on a grid, thereby overcoming the limitations of the Monte Carlo method used in Part I.

The price is that we have to neglect the lateral displacement of the beam, which is correlated with the reflection order, which is correlated with the directional spread. At least in the above-mentioned application scenario (graphite deflector in a neutron spectrometer, far from grazing incidence), this is harmless: the mean lateral displacement is at most a low multiple of the crystal thickness, which is a few millimetres, and therefore corresponds to a fraction of a degree at the next optical element, located 2 m downstream, whereas the deflector crystals have a rocking width of several degrees.

### 2.2. Transport equation and boundary conditions   

The flux obeys the transport equation (Sears, 1989 ▸, equation 8.1.24),
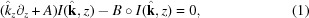
a stationary Boltzmann equation with drift and scattering terms. The linear operator *B* describes gains by Bragg diffraction, 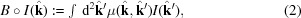
where 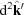 is the solid-angle differential associated with the integration variable 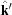. The kernel μ is reviewed below in Section 2.3. The attenuation operator *A* is a multiplicative factor, 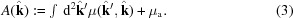
The integral accounts for losses by diffraction. The constant 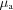 stands for absorption, inelastic scattering, diffuse scattering and diffraction by parasitic reflections (Dorner & Kollmar, 1974 ▸).

To specify boundary conditions, we consider an infinite plate of thickness *d*, extending from 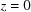 to 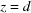. The incident flux 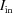 comes from the half space *z* < 0. Accordingly, the boundary conditions are
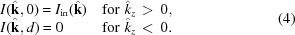
Our task is to compute the reflected and the transmitted flux 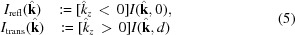
with the indicator function [true] = 1, [false] = 0.

In Part I, we had divided 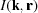 into two functions, 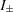, representing the forward and backward traveling beam. Accordingly, the transport equation consisted of two coupled differential equations, generalizing the two-ray Darwin–Hamilton equations of the conventional planar approximation. That notation was useful for describing the reflection-order expansion [Section 3.2 of Part I; see also Grabcev & Stoica (1980 ▸)] as a zigzag walk (Wuttke, 2014*b* ▸) with a strictly alternating sign of 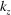. To distinguish two beams we had to exclude the case of grazing incidence. The notation (1), with just one function *I* defined for all 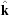, is simpler, more generic and more convenient for our present purpose. Only later, when we choose a grid in 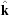 to approximate *I* by a histogram, will we take into consideration the effective two-beam geometry.

### 2.3. Reflection kernel   

The transport kernel in (2) is a *transfer function* that gives the probability per unit length 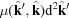 for a particle with incident direction 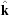 to be scattered into an infinitesimal solid angle 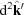 around the outgoing direction 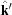. It is a sum 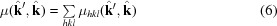
over single-reflection transfer functions, given by an integral 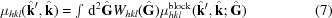
over the block transfer function 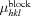 (47) derived in Section A1. The scattering directions 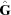 depend on the block orientations, and have the statistical distribution 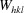.

In certain situations, multiple diffraction by multiple Bragg reflections can be of practical importance (Ohmasa *et al.*, 2016 ▸). Nonetheless, to simplify our exposition, we shall consider only one pair of reflections, *hkl* and 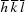. With the joint distribution 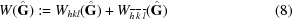
we can merge (6) and (7) into the total transfer function 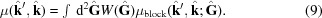
An integration, explained in Section A2, reduces the total transfer function (9) to
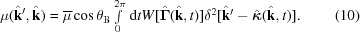
This simplifies in several ways equation I,25 [denoting equation number (25) in Part I], as discussed in Section A3. We now define the symbols 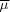, 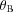, 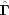 and 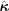 introduced with (10).

The prefactor 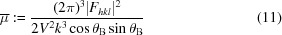
depends on the unit-cell volume *V* and structure factor 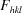. It has the dimension of an absorption coefficient, *i.e.* inverse length. The Bragg angle 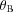 is constant because we consider a fixed reflection *hkl* and a constant radiation wavenumber *k*. Both 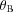 and 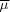 are independent of the sign of the reflection. The outgoing beam direction 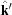 is given by the deflection function 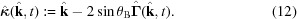
The parametric curve 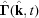 with 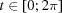 contains all possible scattering directions 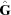 that satisfy the Laue–Bragg condition 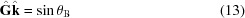
for an incoming wave direction 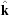.

To construct 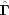, we choose an orthonormal base 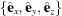 for the reciprocal-space vectors 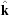 and 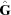. Note that 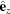 is not required to coincide with the plate normal 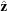 (though it does so in our code and our worked-out examples). Choose a rotation matrix 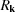 so that 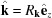 (for readability, we omit carets in subscripts). The circle of possible scattering directions 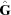 can then be written 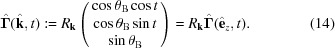
It is straightforward to verify that 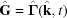, for all *t*, satisfies (13).

The condition 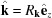 leaves 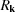 underdetermined, allowing for an arbitrary rotation around 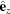. This is irrelevant because the origin of the polar coordinate *t* is arbitrary, and 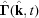 only appears under integrals that run from 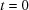 to 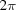.

### 2.4. Specializing the distribution of scattering directions   

For most mosaic crystals, *W* is *isotropic*, *i.e.* invariant under rotation around the mean block normal 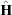. Thereby 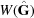 depends only on 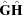.

All the following theoretical developments, including the numeric methodology of Section 4, are independent of what isotropic distribution we choose for 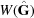. For our numeric examples, however, we need to be more specific. In certain cases (Ohmasa & Chiba, 2018 ▸), *W* can be a ring-like distribution. Here we concentrate on 00*l* reflections, where scattering vectors are parallel to the block normals so that 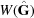 is a disc-like distribution. As is customary, we will choose a Mises–Fisher (MF) distribution (a Gaussian on the unit sphere), 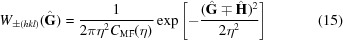
with the normalization constant 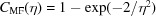. Usually, there is negligible overlap between 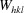 and 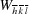 so that the sum (8) can be approximated as 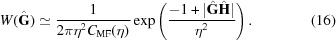
A mosaic with 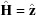 shall be called *normal oriented*. Some consequences of the rotational symmetry around 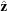 are discussed in Appendix *C* [App appc].

**Figure 2:**
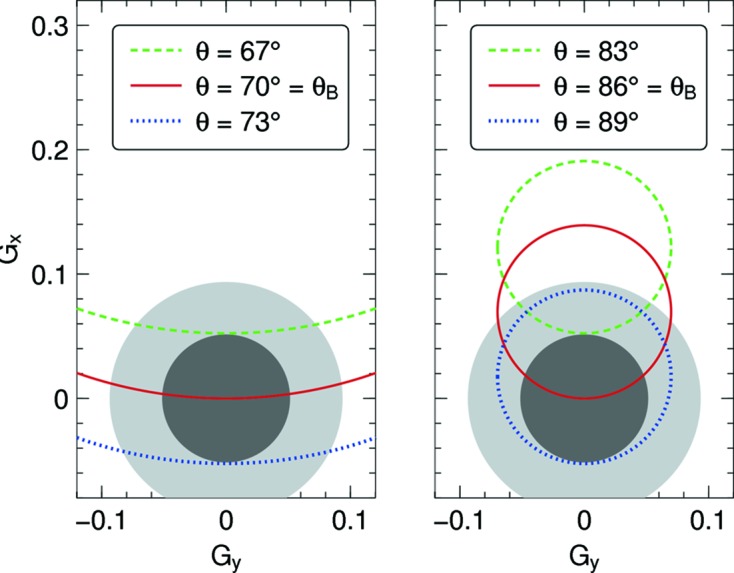
Crystal orientations 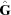 that fulfill the Laue–Bragg condition, projected into the 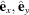 plane, form ellipses. The two plots have different Bragg angles 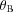. Each plot shows ellipses for three different incident angles 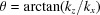, with 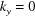. The concentric gray discs contain 50% and 90% of all mosaic blocks, assuming a Mises–Fisher distribution 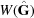 that is centered around 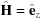, with standard deviation η = 2.5°.

In all numeric examples we assume an isotropic, normal oriented mosaic, and we choose 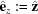. Unless differently stated, the standard deviation is η = 2.5°. The orthographic projection of the circle 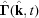 into the 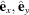 plane is an ellipse. Fig. 2 ▸ shows examples and puts them in relation to 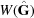.

### 2.5. Parameterization   

In our numeric examples, we will characterize crystals by two dimensionless constants that have a simple intuitive meaning in the two-ray limit (68) for a collimated beam with incident angle 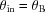. The first of these parameters is the *opacity*

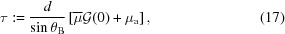
where 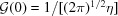. The second dimensionless crystal parameter is the *relative reflectivity*

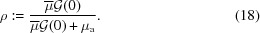
These parameters will enter the following derivations through the products 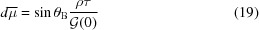
and 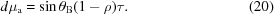
Note that 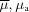 are not pure material constants but also depend on the wavelength of the used radiation. So, in principle, one could tune 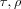 to almost arbitrary values by suitable combinations of wavelength and crystal thickness.

## Discretization in 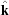   

3.

### 3.1. Binning   

So far, we have assumed that 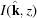 as a function of 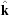 is a distribution on the unit sphere. We now request that the relevant regions of the unit sphere be partitioned in *M* bins, and we replace 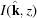 by *M* histograms 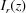 with 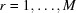. Each histogram represents a current that is defined as flux integral over the solid angle 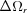, 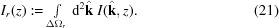
Combining this with the definition (2) of the operator *B*, we get
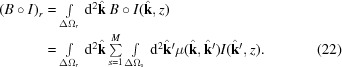
We assume that μ is a sufficiently smooth function of 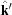 so that it can be drawn in front of the second integral. We obtain 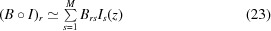
with 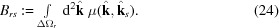
The attenuation factor, discretized in analogy with (24), is 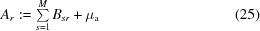
and the transport equation (1) takes the form 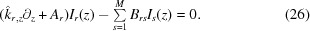
In this paper, we will not investigate errors introduced by the approximation (23). It is up to practitioners to choose appropriate histogram grids so that both discretization errors and computing time be kept within reasonable bounds.

### 3.2. Grids in zero, one, two dimensions   

In Section 4, spectral collocation will be introduced without reference to a particular histogram grid. For our numeric examples, we choose three different grids.

The smallest meaningful grid consists just of 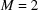 bins, representing a forward and a backward traveling beam, with 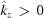 and 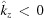, respectively. It will be used in Figs. 5 and 6 to illustrate our approach in the simplest possible way, and to allow verification against the known analytical solution. Instead of the indices *r* = 1, 2, we will use the signs ± to denote the beam direction.

If we are only interested in the total intensity or in the polar distribution of radiation reflected or transmitted by an isotropic, normal oriented mosaic, as in Figs. 9, 10, 12, then we can take an azimuthal average (Section C3), and solve the transport problem on a fine-grained one-dimensional grid in θ.

In all other cases, we need a two-dimensional partition of the unit sphere. Any possible map projection and coordinate system can be chosen to construct the grid. In our examples, we want to preserve the symmetry of the isotropic, normal oriented mosaic and therefore choose a rectangular grid in the spherical coordinates θ and φ.

The one- or two-dimensional grids must not necessarily cover the entire unit sphere. For computational efficiency, we restrict them to two contiguous regions around the transmitted and the reflected beam. These regions can be iteratively adapted, keeping the cut-off error (estimated from the loss channel b, Section B2) under a given tolerance 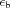 (Section 4.6).

### 3.3. Diffraction matrix   

The integral (24) can be carried out at once since the kernel 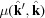, given by (10), contains a delta function. The result is 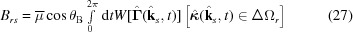
with the indicator bracket as introduced in Section 2.2.

**Figure 3:**
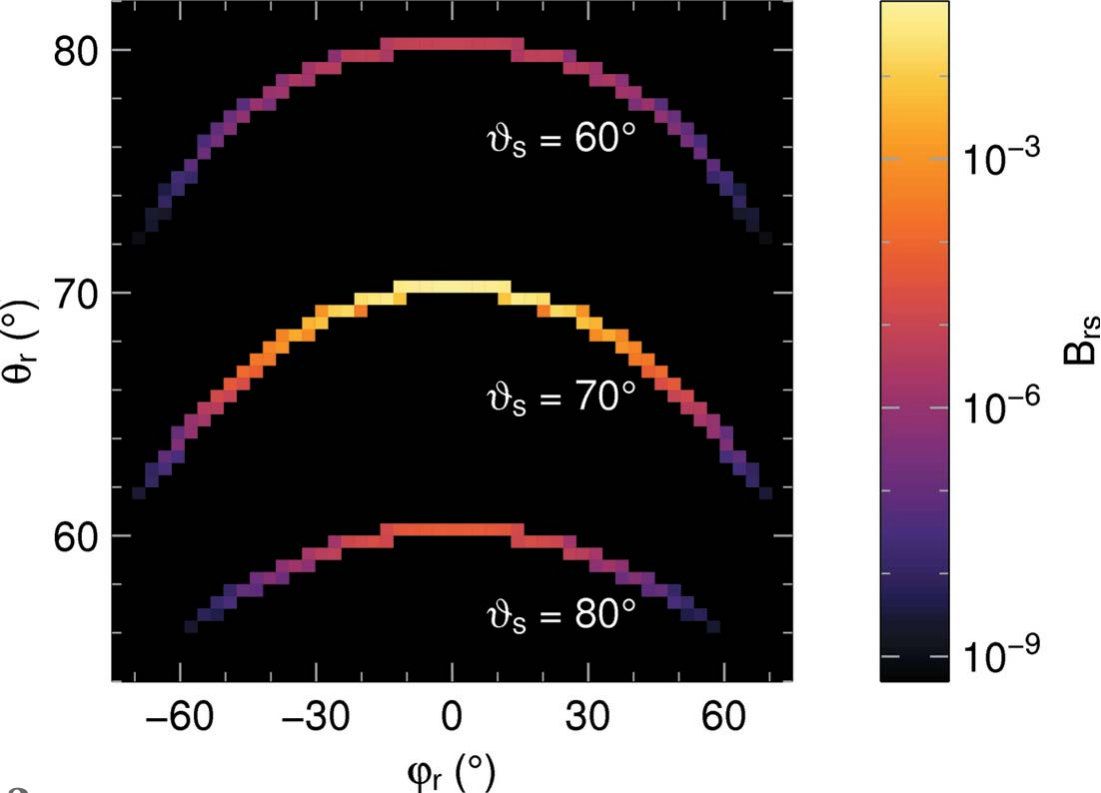
The three bands represent three columns of the reduced reflectivity matrix 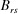, with 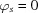 and with three different values of 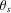, shown as a function of 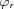 and 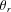. The Bragg angle is 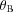 = 70°. There are 80 φ bins from 55° to 85°, and 180 θ bins from −180° to 180°. The dimensionless intensity scale applies for 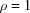 and 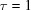.

For given 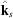, and sweeping *t*, the outgoing directions 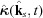 form a one-dimensional manifold on the two-dimensional sphere. This is illustrated in Fig. 3 ▸, which shows these manifolds for three different *s*. In consequence, for a two-dimensional histogram grid, the matrix *B* is *sparse*: most entries are zero, the more so the finer the grid. If each of the two coordinate axes is divided into 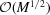 bins, then *B* has 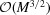 nonzero entries.

Section B1 presents an algorithm for the actual computation of (27). In Section B2, the *M* directional bins are extended by three loss bins. One of them accounts for absorption; the other two allow us to quantify unphysical losses originating from numeric cut-offs. Therefore we can detect violations of particle conservation, and quantify, and ultimately control, numeric approximation errors.

## 4. Spectral collocation in the depth coordinate   

### 4.1. Depth rescaling   

As we will use Chebyshev polynomials in the depth coordinate, it shall be transformed from 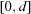 to the standard range 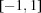. We therefore introduce the reduced coordinate 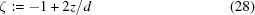
and the transformed histograms
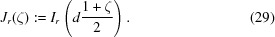
The transport equation (26) becomes 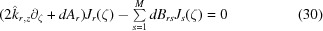
with the separated boundary conditions 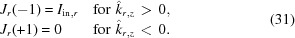
Grids should be constructed such that no bin crosses the equator of the unit sphere.

### 4.2. Equation system   

The equation system (30) consists of *M* coupled first-order linear differential equations in 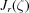. While a formal solution can easily be written down as a matrix exponential, it is numerically not viable (Moler & Loan, 1978 ▸, 2003 ▸). The method of choice for this kind of problem is *spectral collocation*; it is based on the expectation that the solution is a smooth function of ζ and therefore can be expanded in Chebyshev polynomials (Gottlieb *et al.*, 1984 ▸; Canuto *et al.*, 1988 ▸; Trefethen, 2000 ▸).

We approximate the functions 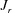 by polynomials 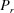 of order *N* that match 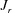 in 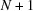 *collocation points* 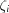. We defer the choice of 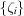 to Section 4.3; until then, we only require 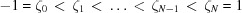. Function values at the collocation points are abbreviated 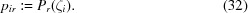
These are 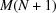 unknowns. *M* of them can immediately be read off from the boundary conditions (31): 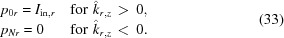
The others will be obtained from the transport equation (30). To discretize this differential equation, we replace 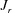 by 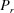, 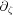 by a differentiation matrix *D*, specified by the requirement
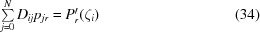
[for an introduction to differentiation matrices, see Trefethen (2000 ▸)]. The resulting *MN* equations
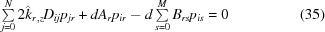
must hold for all histogram bins (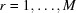) and in all collocation points (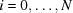).

We collect all linear operators in the matrix 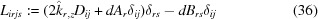
with the Kronecker delta 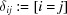 so that the transport equation (35) becomes simply 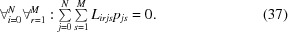
These are 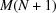 equations in 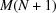 variables 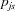, of which *M* are known from (33).

**Figure 4:**
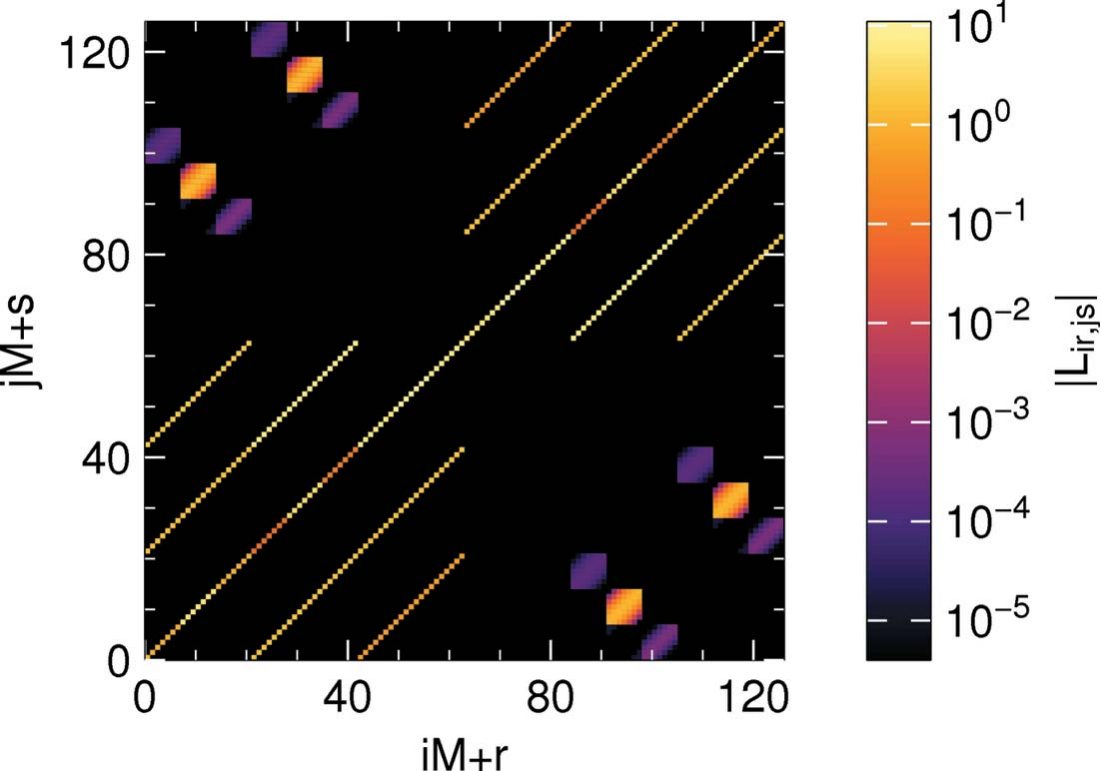
Visualization of a small part of a small matrix *L*. Parameters: 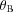 = 70°, 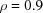, 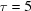. Discretization: three collocation points in *z*; three bins for 55° ≤ θ ≤ 85°; 36 bins for −180° ≤ φ ≤ 180°. Only the 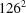 entries with 0 ≤ φ ≤ 60° are shown. Since some entries are negative, the figure shows absolute values 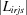.

The matrix *L* is *sparse* because of the Kronecker deltas in its definition (36) and because its component 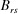 is also sparse when it matters, namely in the computing-intensive case of a two-dimensional grid. In that case, per Section 3.3, of the 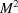 entries of matrix *B*, only 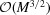 are nonzero. Overall, of the 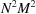 entries of *L*, only 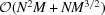 are nonzero. This is visualized in Fig. 4 ▸.

In view of the boundary conditions (4), we now distinguish between histogram bins with *forward* and *backward* propagation direction, according to the sign of 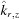. We split the sum over *s* in (37) accordingly, omitting the zero terms in 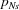 with backward *s*, and bringing the nonzero terms in 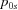 with forward *s* to the right side: 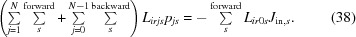
This system of 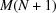 inhomogeneous linear equations in *MN* unknown 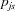 is overdetermined, due to the loss of information that goes along with the reduction of polynomial order in differentiation.

Overdetermination can in principle be avoided by using a rectangular differentiation matrix (Driscoll & Hale, 2016 ▸; Xu & Hale, 2016 ▸). However, this would be unsuitable for the full multi-ray problem because the necessary ‘downcasting’ interpolation of linear terms would make the matrix *L* much less sparse. We rather opt for the standard procedure of simply ignoring redundant equations. We choose to delete the *M* equations with 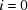, *r* 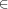 forward or 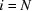, *r* 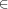 backward.

### 4.3. Collocation points and differentiation matrix   

All of Section 4.2 holds regardless of the collocation points. Their choice, however, is of critical importance for the resulting convergence. We make the standard choice of Chebyshev–Gauss–Lobatto points, which are located at the extrema of the Chebyshev polynomial 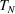, 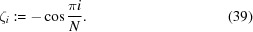
They only enter our computation through the corresponding differentiation matrix (34).

This matrix has the outer diagonal entries 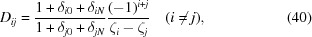 the interior diagonal entries 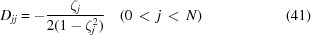 and the diagonal endpoints 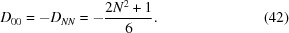
For a derivation, see *e.g.* Gottlieb *et al.* (1984 ▸) or Trefethen (2000 ▸), but note that our choice of ascending 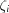 has led to a minus sign on the right-hand side of (42).

### 4.4. Collocation error for the two-ray reference   

By the *collocation error* we understand the error caused by approximating the functions 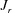 by polynomials 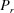. We first consider the two-ray approximation (re-derived in Section D1) for which we can determine the collocation error by comparing with the known analytical results (summarized in Section D2). We choose 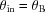 so that the Bragg operator (68) is simply 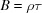 and the total attenuation 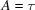.

**Figure 5:**
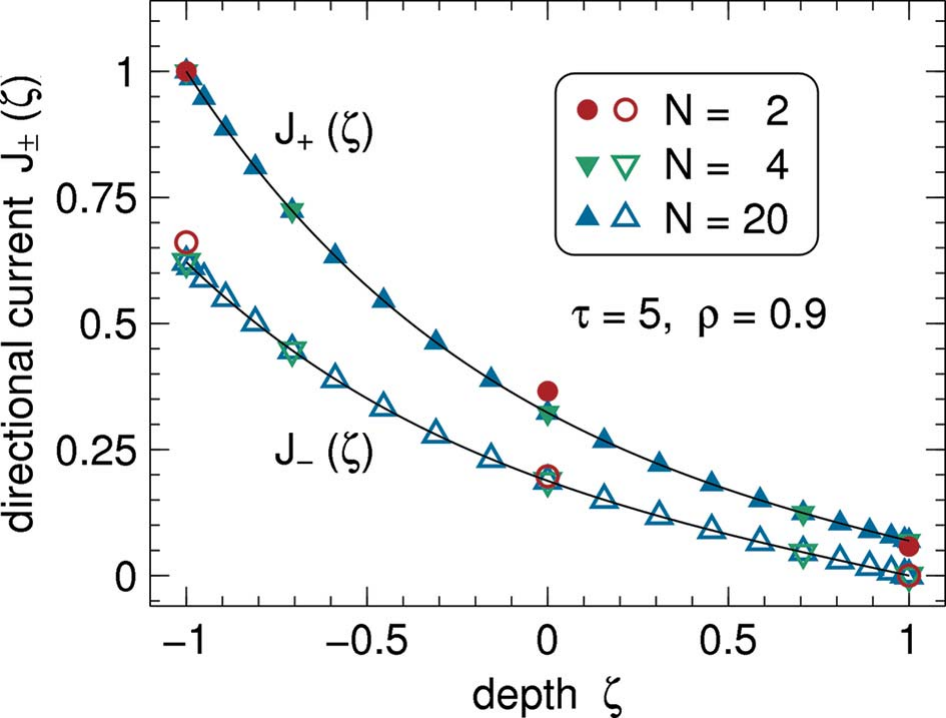
Directional currents (29) as a function of depth for 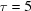, 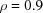. Lines show the analytical solution (70). Symbols have been computed by spectral collocation with different *N*.

Fig. 5 ▸ shows currents 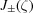 versus ζ for a moderately thick crystal with realistic attenuation: 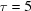, 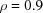. On the linear scale of this plot, the numeric data points and the analytical curves (70) agree perfectly for collocation orders as low as 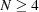.

**Figure 6:**
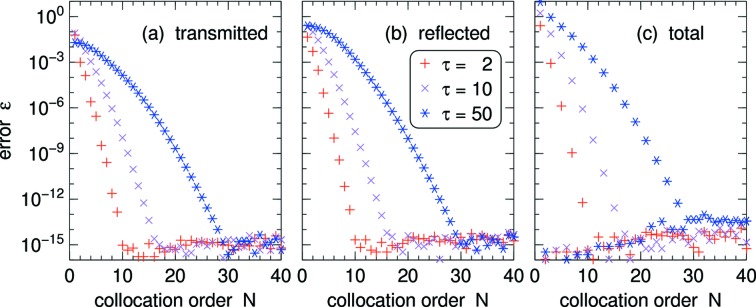
Accuracy of spectral collocation as a function of the number of collocation points *N*, for the planar two-ray model, for different crystal opacities. (*a*), (*b*) Absolute error of the transmitted and reflected current, determined by comparison with the analytical result (71). (*c*) Deviation of the total current (transmitted, reflected and absorbed) from the true value 1.

The rapid convergence of the spectral collocation is further demonstrated in Fig. 6 ▸, which shows the deviation from the analytical result as a function of *N*. The decrease of the error with increasing *N* is roughly exponential until some base level is reached.

Note that the figure represents the *absolute* error. This could become a problem in shielding calculations for very thick mosaic crystals where a tiny transmittivity would result in an unacceptable *relative* error. We exclude this peculiar case from further consideration.

Fig. 6 ▸(*c*) shows the error of the total current 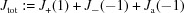 where 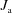 is the absorption loss channel (Section B2). Analytically, the true value is 1. For odd *N*, convergence takes about as long as in Figs. 6 ▸(*a*), 6 ▸(*b*). In contrast, even for the smallest even *N* the error is only of the order of machine precision, thanks to pairwise cancelation at collocation points *i*, 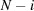. Therefore we generally prefer even *N* in our computations.

### 4.5. Collocation error for the full model   

We now investigate the collocation error for a two-dimensional 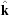 grid, for representative crystal parameters and for an ideal collimated incoming beam 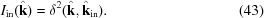
We only consider the reflected flux 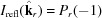.

**Figure 7:**
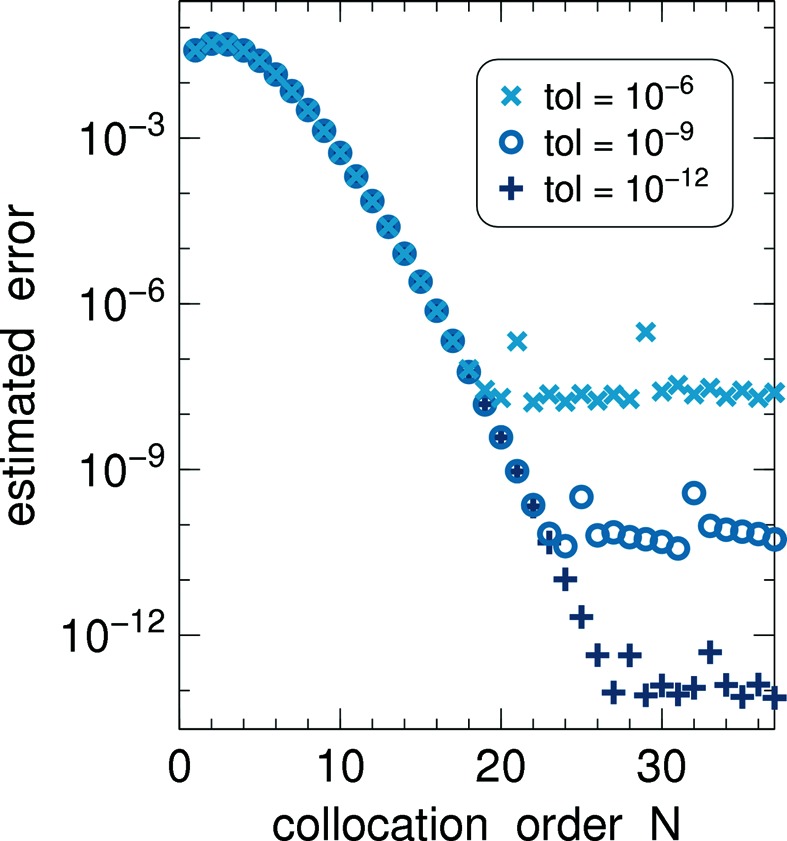
Estimates of the collocation error 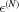 of the reflected current 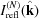 as a function of the collocation order *N*, for different solver convergence tolerances. Model parameters 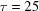, 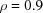, 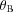 = 70°. Collimated incoming beam (43) with 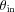 = 70°. Discretization: 45 bins for 58° ≤ θ ≤ 82°; 11 bins for −180° ≤ φ ≤ 180°.

In contrast to Section 4.4, no analytical solution is known. Therefore we estimate the overall collocation error by comparing approximate solutions at successive collocation orders *N*, 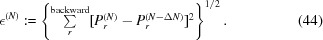
In Figs. 7 ▸ and 8 ▸, we increase *N* from 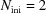 in steps of 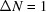; elsewhere, larger values of the hyperparameters 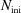 and 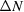 are more convenient (Section 4.6).

In Fig. 7 ▸, the error estimate 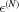 is shown as a function of *N* for different solver tolerances. The behavior is known from Fig. 6 ▸(*b*): after a roughly exponential decrease the 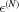 cross over to a noisy base level that lies safely below the solver tolerance.

**Figure 8:**
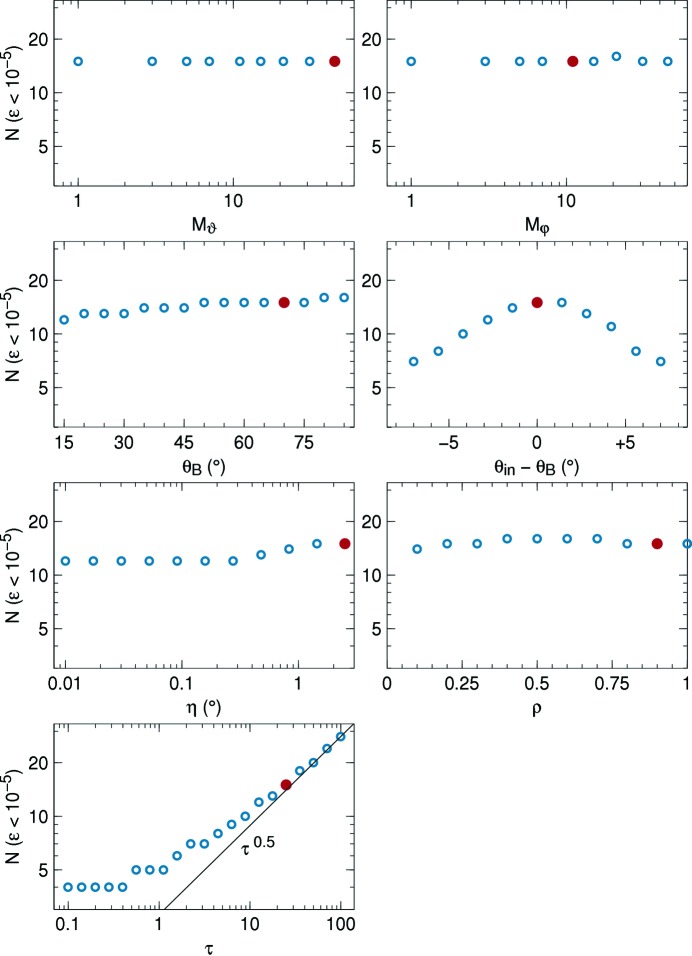
Minimal collocation order *N* required to keep the overall error (44) of the reflected flux below 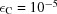. In each graph, one of the parameters of the default model of Fig. 7 ▸ is varied. In each graph, a red disc indicates the parameter value that is used in all other graphs.

The collocation order *N* is determined by repeating the entire computation for increasing values of *N* until 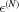 lies below a given bound 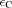 (Section 4.6). Fig. 8 ▸ shows how the so-determined minimal collocation order *N* varies with the grid size 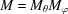 and with the model parameters 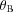, 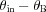, η, ρ, τ. All these parameters are found to be uncritical, except the opacity τ, for which Fig. 8 ▸ suggests a possible asymptote 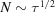. Therefore our computational method should not be applied to extremely thick crystals with 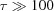. This is of no concern for reflectivity computations: it is always possible to restrict τ to values of about 20 or 30; anything beyond is inconsequential for the reflected current.

### 4.6. Hyperparameters and *a posteriori* tolerances   

The numeric solution is controlled by *hyperparameters* (so-called in opposition to the regular parameters that describe the crystal model):

(i) Bounds in θ and φ, and numbers of bins, to specify the directional grid.

(ii) Initial number of collocation points and increment for the iterative procedure described in the last paragraph of Section 4.5. Our default choice is 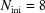 and 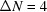.

(iii) Auxiliary parameters for the numeric evaluation of the reflection kernel (27), as explained in Section B1: bisection accuracy 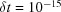, maximum step size 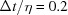 and a kernel cut-off 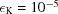.

(iv) The *collocation tolerance* 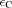 (Section 4.5): the sparse equation solver is called with increasing *N* until the estimated error 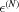 [equation (44)] falls below 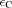. We use 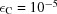.

(v) The *solver tolerance* 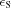 required by the sparse equation solver (Section 4.7). It should be smaller than 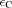. We use a value of 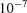, except when we determine collocation errors as a function of the number of collocation points: Fig. 6 ▸ is computed with 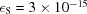 and Fig. 7 ▸ with 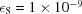.

Additionally, some *a posteriori* tolerances are used in checking the numeric integrity of the obtained solution. If any check fails, then we recompute everything with stricter hyperparameters. These tolerances are:

(i) A *noise level* 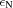 to disallow currents below 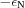, as may be caused by numeric inaccuracies at weak intensities.

(ii) A stricter limit 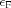 is imposed to the absolute value of the total current (53).

(iii) The *cut-off tolerances* 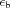 and 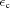 are upper limits for the loss channels 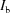 and 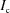 that account for unphysical losses due to the finite 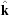 grid and the deletion of weak matrix elements (Section B2).

All our numeric examples have been checked with 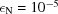 and 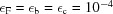.

### 4.7. Numeric tools   

A sparse LU solver is used to invert the remaining *MN* equations. Our implementation is based on tools from the numerical library *Trilinos* (Heroux *et al.*, 2003 ▸), namely the sparse compressed row matrix class from package *Epetra*, the ILU(0) *preconditioner* from package *Ifpack* (Sala & Heroux, 2005 ▸) and the GMRES (generalized minimal residual) *block solver* from package *Belos* (Bavier *et al.*, 2012 ▸). The mapping of array indices is described in the supporting information.

## 5. Results   

### 5.1. Open-source code *MultiBragg*   

The software developed along with this work is released under the GNU Public License (GPL v3 or higher), and deposited in the form of a compressed tar.gz archive as supporting information. The code comprises a library *MultiBragg* for the numeric solution of the transport equation, and application programs that generate the data for the figures in this work. More information on the software is provided in the textual part of the supporting information.

### 5.2. Total reflectivity   

**Figure 9:**
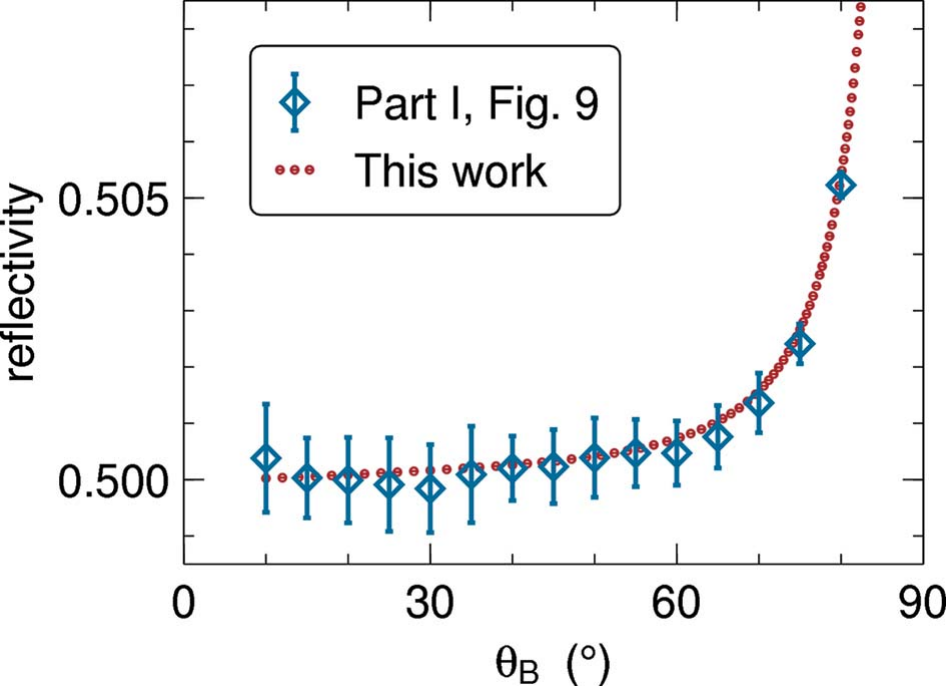
Total reflectivity as in case 1 of Fig. 9 of Part I. Collimated incoming beam with 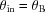; mosaicity 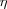 = 0.025 rad; nominal opacity 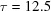; relative reflectivity 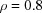. In terms of Part I: 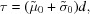 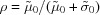. Blue symbols with error bars are from the Monte Carlo computation of Part I; red circles are from the present numeric integration.

**Figure 10:**
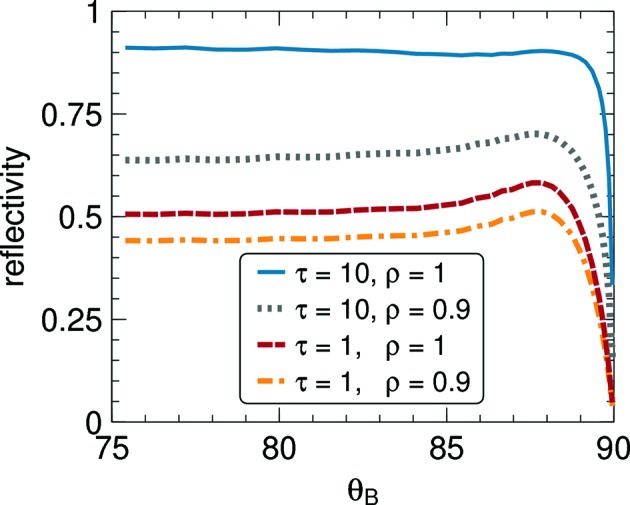
Total reflectivity near backscattering, for a collimated incoming beam with 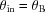; mosaicity η = 2.5°.

Fig. 9 of Part I showed the total reflectivity *R* for plates of different opacities as a function of the Bragg angle 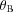, computed by Monte Carlo integration. The present Fig. 9 ▸ compares old and new results. The perfect accord of both data sets provides strong support for the correctness of both computer codes. While error bars in Part I were of the order 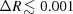, our new results are far more accurate and extend over a wider 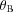 range. Our new method is also much faster: computing times are typically of the order of some minutes, and only of a few seconds if no azimuthal resolution is needed, as here and in Figs. 10 and 12.

In the 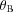 range covered by Part I, corrections from non-planarity amounted to 1% at most. Our numeric method now allows us to compute *R* up to 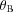 = 90°. Representative results are shown in Fig. 10 ▸. With 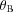 approaching 90°, the reflectivity first increases, then decreases rapidly towards 0. These observations are easily understood by looking back to Fig. 2 ▸: close to backscattering, the ellipse of block orientations 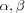 that fulfill the Bragg condition is almost a circle, and for 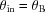 it is concentric with the disc representing 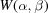. This makes it plausible that the reflectivity can rise more than 15% above the constant value from planar theory. Even closer to backscattering, however, the ellipse shrinks towards a point, fewer blocks are available for Bragg diffraction, and the reflectivity decreases proportionally.

### 5.3. Azimuthal distribution   

**Figure 11:**
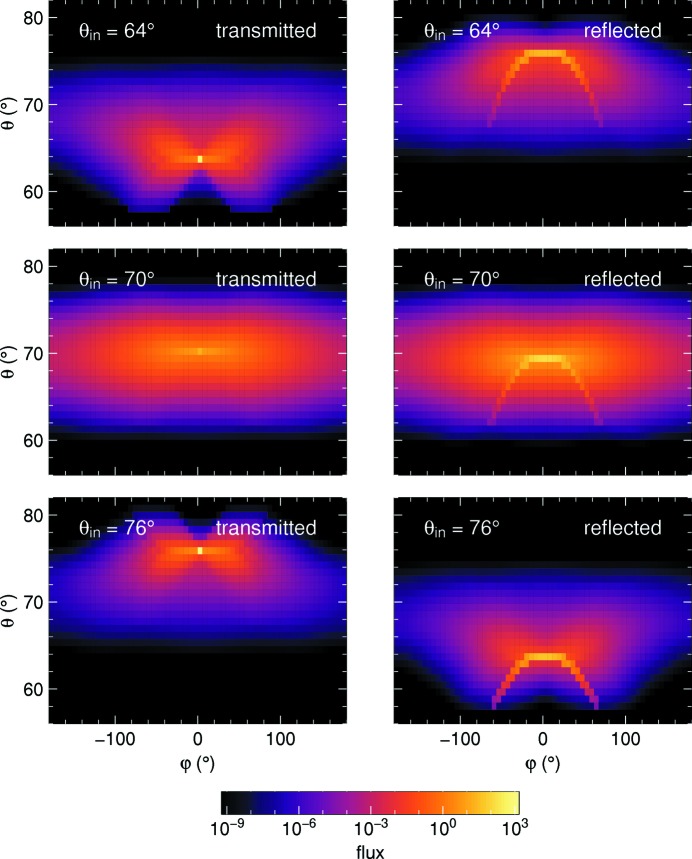
Directional distribution of the transmitted and reflected flux for three different inclinations 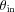 of the incoming collimated beam. Crystal parameters: 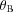 = 70°, η = 2.5°, 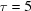, 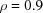.

Fig. 11 ▸ shows the directional distribution of the transmitted and reflected radiation for three different incoming beam inclinations 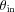. Since the coordinate representation does not account for the different bin sizes 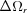, this figure does not show currents (flux integrals per bin) but the directional flux 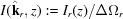.

In transmission, a bright spot shows the attenuated incoming beam. It is least pronounced at 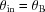, where the reflectivity is strongest. In the reflected distribution, the parabolic trace comes from one-reflection trajectories, whereas the diffuse cloud represents the sum of all higher reflection orders. While the parabola is known from the approximative treatment of Hennig *et al.* (2011 ▸), the two-dimensional cloud is only accounted for by the full transport equation solved here.

**Figure 12:**
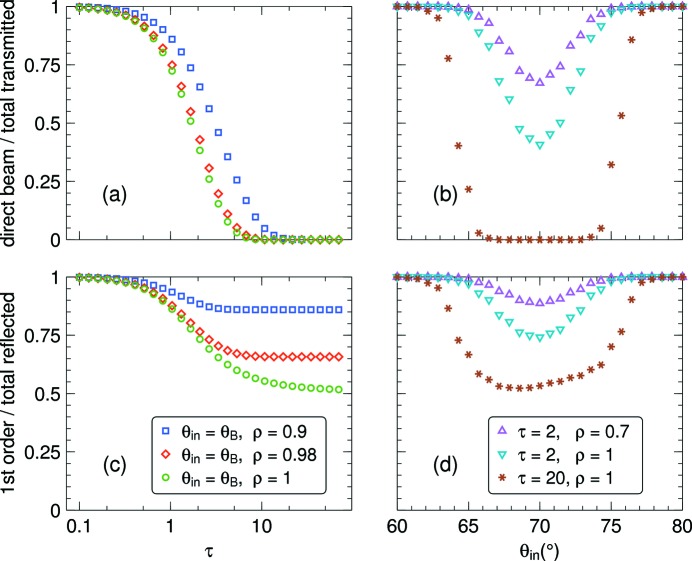
(*a*), (*b*) Relative contribution of the direct beam to the total transmitted intensity; (*c*), (*d*) relative contribution of single reflections to the total reflected intensity. All data for 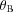 = 70°; (*a*), (*c*) as a function of τ for different values of ρ, with 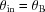; (*b*), (*d*) as a function of 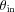 for different combinations of τ and ρ.

To visualize the relative importance of this cloud, Fig. 12 ▸ shows the intensity of the direct beam and the single-reflected spray relative to the total transmitted or reflected intensity. Multiple reflections are most important for high opacity τ, high relative reflectivity ρ, and for incident beam directions close to the Bragg condition, 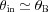. For 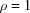 and 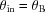, the relative importance of direct transmission goes quickly to 0 for increasing τ. The relative importance of single reflections decreases more slowly, and the limit of 50% is not fully attained within the τ range of the figure.

### 5.4. Rocking curves   

The standard way to characterize a mosaic crystal experimentally is by measuring a *rocking curve* (*e.g.* Schneider, 1974 ▸): the reflected or transmitted intensity is recorded while the crystal is rotated around an axis normal to the scattering plane. In our fixed-crystal frame, this is equivalent to scanning the incident angle 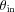 while maintaining the detector angle at 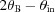.

**Figure 13:**
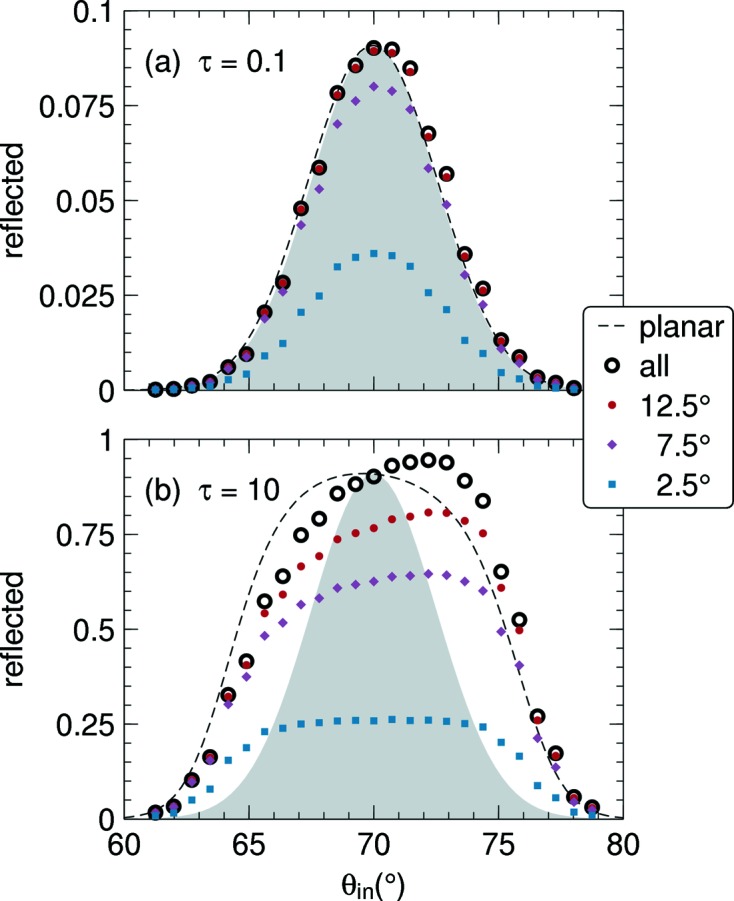
Rocking curves for (*a*) a thin, (*b*) a thick mosaic crystal with 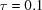 and 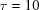, respectively. The gray area indicates the Gaussian mosaic distribution with standard variation η = 2.5° as assumed throughout this work. The dashed line is the reflectivity in planar approximation (71). Symbols represent numeric solutions of the full transport equation: small colored symbols show the intensity collected by circular detectors with radius specified as an angle; thick black circles show the entire reflected radiation.

It is well known from theory and experiment (Dorner & Kollmar, 1974 ▸) that rocking curves are generally wider than the underlying crystallite orientation distribution 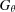. The width increases with increasing opacity τ. This is illustrated by Fig. 13 ▸ where rocking curves for a very thin (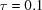) and a very thick (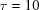) mosaic are shown. In the thin-crystal limit, our solution of the full Darwin–Hamilton equations reproduces Sears’ solution of the planar approximation (71), and both curves coincide almost perfectly with the Gaussian 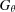.

Conversely, for the thick crystal Sears’ solution is much wider than 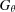, and our full solution deviates from Sears’ in that it is shifted by about 1° and has a slight asymmetry. This confirms the Monte Carlo result of Fig. I,10, and extends it to Bragg angles further away from backscattering.

So far, we have discussed total reflected intensities. In practice, detectors cover only a finite solid angle. Rocking curves as measured by circular detectors are shown by the colored open symbols in Fig. 13 ▸. Unless the opening angle is considerably larger than η a considerable part of the reflected intensity is indeed lost outside the detector. For the thick crystal, the shape of the rocking curve also varies considerably with the angular coverage.

## 6. Conclusions   

To summarize, we have simplified the transport equation of Part I (Wuttke, 2014*a* ▸) by making consequential use of energy conservation and projecting everything to the sphere 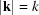.

For isotropic, normal oriented mosaics (Section 2.4), azimuthal current distributions are insensitive to the resolution in the polar angle φ; if the polar distribution does not matter, then numeric computations can be accelerated by considering one single φ bin (Sections 3.2, C3). From there, only one more linearization (Section D1) is needed to explain how the original planar Darwin–Hamilton equations got the integral currents essentially right.

Our first numeric result (Section 5.2, Fig. 9 ▸) confirms that off-plane trajectories have very little effect upon the integral currents except near backscattering. The interest of our present work is not in those minor corrections, but in deriving information that is not at all available from the original Darwin–Hamilton equations, namely the directional distribution of the transmitted and reflected radiation.

While Wuttke (2014*a* ▸) presented some asymptotic results, a formal expansion in reflection order and a Monte Carlo code, we now have derived a numeric scheme that uses spectral collocation in the depth coordinate to compute 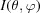 with high speed and very high accuracy. Fig. 11 ▸ shows an example outcome: a dot represents the direct beam, a parabolic spray comes from single reflections, whereas all higher reflection orders contribute to a diffuse, two-dimensional cloud of propagation directions. Fig. 13 ▸ shows the consequences for rocking-curve measurements.

As mosaic crystals are an important beam optical device, numeric solutions of the transport equation will help to improve instrument and radiation protection simulations. The computer code produced for this work is open source and freely available, and will hopefully find its way into established ray-tracing packages.

## Supplementary Material

Open-source software MultiBragg as used to generate Figs 3-13. DOI: 10.1107/S2053273320002065/ae5082sup1.gz

## Figures and Tables

**Table 1:** Correspondence of notations in Sears (1997 ▸) and in the present work

Variable	Sears (1997 ▸)	This work
Crystal thickness	*d*	*d*
Depth variable	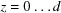	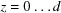
Rescaled depth variable		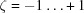
Bragg angle	θ	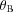
Forward current	*I*	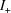
Backward current	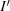	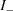
Forward beam polar angle	φ	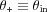
Backward beam polar angle	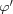	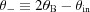
Bragg cross section	σ	*B*
Bragg cross section, reduced, forward beam	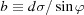	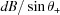
Bragg cross section, reduced, backward beam	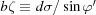	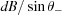
Loss cross section	μ	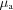
Total attenuation cross section	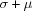	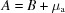
Total attenuation cross section, reduced, forward beam	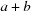	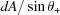
Total attenuation cross section, reduced, backward beam	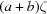	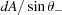

## Appendix A Transfer function

### A1. Block transfer function

In Part I, the transfer function of a single-crystalline block was derived in three-dimensional wavevector space (equation I,12),

The prefactor , introduced in (11), agrees with equations I,7 and I,26, with equation 53 in Sears (1997 ▸), and equation A7 in Grabcev & Stoica (1980 ▸). The three-dimensional delta function in (45) can be decomposed as

where  is the delta function on the unit sphere. The scalar delta function is implicit in our redefinition of distribution functions, . By comparing the three- and two-dimensional variants of the transport equation we see that it cancels. The factor  cancels when casting the three-dimensional integral (equation I,38) to our two-dimensional definition (2) of the Bragg operator. A factor  can be drawn out of the first delta function in (45). Altogether we obtain

The first delta function enforces the Laue–Bragg condition (13). The second delta function in (47) ensures that the diffracted wave propagates in a direction  given by the deflection function , defined in (12).

### A2. Total transfer function

To carry out the integral (9), we consider how an integral in  acts on the first delta function of (47). We parameterize  in spherical coordinates  with respect to the axis , and introduce a test function *f* to find

The remaining integral involves a full circle in . This circle arises as the intersection of the unit sphere with the plane defined by the Bragg condition (13). For given , we will write this circle as . Because it only appears under integrals running from  to , we can leave the origin in *t* (the orientation of the spherical coordinates around ) unspecified.

### A3. Relation to Part I

The above is a simplification in three ways over Part I (equations 25,27): thanks to the parameterization in *t*, there is no more need to sum over two half  circles (or ellipses, when referring to the orthographic parameterization of Part I). The singularity  in the correction factor *h* has canceled under the substitution , so that there is no longer a singularity near backscattering. And the β dependence in the second fraction in equation I,27 is gone for good as we no longer use the approximation  implicit in equation I,13.

## Appendix B Diffraction matrix

### b1. Numeric computation

To compute the matrix element  for given *s*, we divide the integration domain in (27) in finite intervals. We use bisection to determine points  such that for  all  lie in one and the same bin . If necessary, intervals are further divided to ensure . This simplifies (27) to

Integrals now extend over ranges so small that Gauss–Legendre three-point quadrature is good enough; the accuracy has been ascertained by comparison with higher-order rules. We compute  for the midpoint . From this, we easily deduce  and increment the corresponding . Quite some computational effort can be saved in the special case of an isotropic, normal oriented mosaic, as discussed in Section C2.

To make the matrix *B* sparser, and thereby speed up the solution of the discretized transport equation, we delete matrix entries that are too tiny to have any consequence for the question under study. Specifically, we set  to zero if it is smaller than . Our default choice for the cut-off hyperparameter is  (Section 4.6).

### b2. Loss channels

If , then the total current in the  direction is constant,

This identity can be used to check the accuracy of a numeric solution.

To maintain (50) even in the presence of non-diffractive losses, we increment *M* by 1 to allow for a *loss channel* . Numeric experimentation shows that the preferred propagation direction is backward, say , which implies that the proper boundary condition is . The diffraction matrix acquires the additional entries

and the term  appears no longer explicitly in the attenuation factor (25),

This approach comes to fruition with two more loss channels,  and , which account for errors introduced by two approximations that reduce the number of nonzero matrix entries :

One approximation consists of choosing a grid that does not cover the full unit sphere (Section 3.2). It makes (22) inexact because for some *s* there is a finite probability  of diffraction towards some  that is not in the grid.

The other approximation is the zeroing of matrix entries  that are too tiny to have any practical consequence (Section B1). To assess the overall error made by this approximation, we set  to the sum of all deleted entries  for given *s*.

As per (51), there is no scattering out of these channels: . Starting from , intensity accumulates with decreasing *z*. The total *approximation losses* can be read off from  and . If they are below tolerances  (Section 4.6), then one can be sure that approximations made by restricting the grid and by zeroing some  are unproblematic.

Altogether, the total current, including all loss channels, is

Allowing for some numeric inaccuracy, the absolute value of the left-hand side is requested to stay below a tolerance  (Section 4.6).

## Appendix C Isotropic, normal oriented mosaic

### c1. Spherical coordinates

Isotropic, normal oriented mosaics (defined in Section 2.4) have a rotational symmetry around  that allows us to simplify and accelerate some computations. For given , we choose orthonormal vectors . For a given reciprocal-space direction , we define spherical coordinates  with respect to the base :

We choose the rotation matrix in equation (14) as , where  are rotations around the  axes. It is easily verified that . The deflection function (12) is

With the last line, we obtained a factorization of the  and  dependence of .

### c2. Computing the diffraction matrix

The algorithm to compute the diffraction matrix  described in Section B1 has an outer loop that runs over the bin index . If for an isotropic, normal oriented mosaic bins are chosen on a rectangular grid in the spherical coordinates , then that loop must only be executed for  at one fixed . In a second step, results are then transcribed to all other ; the outgoing bin indices *r* are translated accordingly. This is particularly easy if the φ grid extends over  so that a periodic boundary condition applies. A further factor of 2 in computational effort can be saved by using the symmetry of  and *W* under a change of sign of *t*.

Given the circulancy of *B* in φ, one could also decouple equations by Fourier transform. However, this would come at an expense in sparsity, and it would not help for future applications with non-normal oriented mosaics. Therefore, we have not explored this idea any further.

### c3. Azimuthal average

If we are not interested in the azimuthal distribution of the radiation reflected or transmitted from an isotropic, normal oriented mosaic then the rotational symmetry allows us to integrate out the coordinate φ so that only the θ dependence of the flux

is studied further. To integrate over the transfer function (10), we note that  is independent of . We find the deflection operator (2)

with the transfer function

and the polar deflection function

We integrate (58) to obtain the attenuation operator

with the dimensionless total deflection probability

## Appendix D The two-ray model

### d1. Derivation

If the reflected polar angle (59) is expanded in  and *t*, then the lowest order is just [in accord with equation I,64, but note that the next order has been corrected in Wuttke (2020 ▸)]

Accordingly, (58) and (61) are simplified to

and

An incoming collimated beam, through arbitrarily many reflections, will propagate forward along  and backward along , as described by the two-ray Darwin–Hamilton equations.

Realistic mosaic distributions are narrow; in radians, . This justifies the above truncation, and motivates a series expansion of  in the small arguments  and *t*, for use in (63). In lowest order, we find

Specifically, with the Mises–Fisher distribution (16), we can carry out the integral (64) to find

with the normalized univariate Gaussian

In the two-ray notation of Part I, the Bragg operator (57) becomes

in agreement with the standard treatment of the planar Darwin–Hamilton equations (Zachariasen, 1945 ▸, equation 4.19; Sears, 1989 ▸, equation 5.2.70).

### d2. Analytical solution

The two-ray boundary problem with plate geometry has been solved in full generality by Sears (1997 ▸). Since our notation deviates from Sears’ in potentially confusing ways, translations are given in Table 1 ▸. According to (68), the Bragg cross sections are the same for both rays: . The abbreviations from Sears’ equation 12 are in our notation

The resulting directional currents  are then given by Sears’ equation 15.

We now specialize to the symmetric case , hence , as addressed in Fig. 5 ▸. With our abbreviations τ and ρ from Section 2.5 and with the further abbreviation , (69) reduces to ,  and . Sears’ equation 15 yields

To discuss rocking curves, we need the transmittivity and reflectivity for arbitrary  (Sears’ equation 16)]:

For , we obtain by specializing either (70) or (71)
