# Supplementary material for: Multiple Bragg reflection by a thick mosaic crystal. II. Simplified transport equation solved on a grid
Source: Acta Crystallogr A Found Adv. 2020 Apr 16;76(Pt 3):376–89. doi: 10.1107/S2053273320002065 (PMC7233015; doi:10.1107/S2053273320002065)
Supplement: Supplementary file 1 [file a-76-00376-sup1.gz › Readme.pdf]

Multiple Bragg reflection by a thick mosaic crystal.
II. Simplified transport equation solved on a grid.
Supporting Information: Software MultiBragg

Folkmar Bornemann[1], Yun Yvonna Li[2], and Joachim Wuttke[2]*
[1] *Zentrum Mathematik M3, Technische Universität München, 85747 Garching, Germany and*
[2] *Jülich Centre for Neutron Science at MLZ, Forschungszentrum Jülich GmbH, 85747 Garching, Germany*
(Dated: January 31, 2020)

The software *MultiBragg* solves the transport equation for radiation that is multiply reflected by an ideal imperfect mosaic crystal. The underlying theory and exemplary results are published in [1]. This document provides supplementary information, namely instructions how to build and to use the software, and some algebraic and arithmetic derivations that help to understand the code.

I. INTRODUCTION

This text document `Readme.pdf` is part of the source archive of the software `MultiBragg`. This software has been developed along with our paper "Multiple Bragg reflection by a thick mosaic crystal. II. Simplified transport equation solved on a grid" [1]. It has been used to work out the examples and to generate most figures in that paper. Therefore, this source archive is deposited as Supporting Information at Acta Crystallographica A.

The `MultiBragg` code is released under the GNU Public License (GPL v3 or higher); other licensing is negotiable. One of us (Joachim Wuttke) intends to continue work with and on the code. Therefore, before using the sources from this archive for novel work, it is recommendable to ask him for updates. The main purpose of this archive is to ensure reproducibility of the results reported in the paper.

Sect. II of this document provides information how to build and use the software. Sect. III collects some algebraic and arithmetic derivations that close the gap between the published methodology [1] and the actual implementation.

II. THE SOFTWARE

A. Code structure

The software is written in the programming language `C++17`, and comes with `CMake` build scripts. A few auxiliary scripts are written in `Python`. The bulk of the code is organized in a shared library, `libmultibraggcore`, with sources in directory `lib/`. The application programming interface (API) is declared in file `multibragg.h`. Code documentation can be generated by running *Doxygen* [2] over the source directory.

Usage of the library is demonstrated by the application program `doit` in directory `interactive/`. It reads parameters from the command line, and prints the transmitted and reflected flux.

B. Building the software

This software should work on any platform, but has been tested under Linux only. External requirements are:

- any MPI library, e. g. `Open MPI`,
- the numerical library Trilinos [3] (components Epetra Teuchos TeuchosParameterList Belos Ifpack).

To build out-of-source, go to the top-level source directory and run

```
mkdir build ,
cd build
cmake ..
make
ctest
```

C. Reproducing the figures

Directory `plot/` of this source archive contains application programs `generate_fig...` that generate all the data points shown in Figs. 3–13 of the main paper [1]. These figures were created using our own data analysis and plotting software *Frida* [4], which has the advantage of generating graphics in form of human editable *PostScript* code. Subsequent manual editing is necessary for instance to add legends to the figures.

This manual step, however, breaks automatic reproducibility. To ensure nonetheless that we can at any moment reproduce the data contents of the figures, we implemented the following: The directory `fig/` of this source archive contains a verbatim copy of the final *PostScript* source of Figs. 3–13 as used in producing the published paper [1].

The command `ctest`, besides some unit tests, runs for each figure in `fig/` a regression test `plot/persistence_test_fridaplot.py`. This test regenerates the raw figure from scratch, runs

* j.wuttke@fz-juelich.de

plot/fridaplot2data.py to extract the plot coordinates of all data points from the raw figure and from the manually edited figure, converts them to world coordinates, and runs plot/diffdata.py to ensure that data have not changed within a relative error margin of $3 \cdot 10^{-5}$. This scheme not only ensures the future reproducibility of our results, but also helped us during our research, as it ensured consistency whenever we modified our core software.

For some figures, in routine tests we actually use a reference variant (.ps.ref) that differs from the published figure in that it has less bins, and therefore can be regenerated much faster.

III. DERIVATIONS

In this section, some mathematical results of the main paper [1] are worked out in more detail to facilitate the understanding of the actual implementation in our MultiBragg code. Equation numbers below (72) refer to the main paper.

A. Deflection in spherical coordinates

Since we only consider isotropic, normal oriented mosaics, we work in spherical coordinates (53). To compute the diffraction matrix B_{rs} on a $\theta_{\boldsymbol{k}}, \varphi_{\boldsymbol{k}}$ grid, we need the coordinates $\theta_{\boldsymbol{\kappa}}, \varphi_{\boldsymbol{\kappa}}$ of the deflection function (54).

We start by writing

$$R_{\boldsymbol{y}}(\pi/2-\theta_{\boldsymbol{k}})\hat{\boldsymbol{\Gamma}}(\hat{\boldsymbol{z}},t) = \begin{pmatrix} \alpha(\theta_{\boldsymbol{k}},t) \\ \beta(t) \\ \gamma(\theta_{\boldsymbol{k}},t) \end{pmatrix}, \tag{72}$$

where we introduced three scalar functions in accord with Eq. 23 of Part I [5]:

$$\alpha(\theta,t) := \cos\theta_\mathrm{B}\sin\theta\cos t + \sin\theta_\mathrm{B}\cos\theta, \tag{73}$$

$$\beta(t) := \cos\theta_\mathrm{B}\sin t, \tag{74}$$

$$\gamma(\theta,t) := -\cos\theta_\mathrm{B}\cos\theta\cos t + \sin\theta_\mathrm{B}\sin\theta. \tag{75}$$

This enables us to evaluate (54) as

$$\begin{aligned}\hat{\boldsymbol{\kappa}}(\hat{\boldsymbol{k}},t) &= R_{\boldsymbol{z}}(\varphi_{\boldsymbol{k}})R_{\boldsymbol{y}}(\pi/2-\theta_{\boldsymbol{k}})\left\{\hat{\boldsymbol{z}} - 2\sin\theta_\mathrm{B}\hat{\boldsymbol{\Gamma}}(\hat{\boldsymbol{z}},t)\right\} \\ &= R_{\boldsymbol{z}}(\varphi_{\boldsymbol{k}})\begin{pmatrix} \cos\theta_{\boldsymbol{k}} - 2\sin\theta_\mathrm{B}\,\alpha(\theta_{\boldsymbol{k}},t) \\ 0 \quad - 2\sin\theta_\mathrm{B}\,\beta(t) \\ \sin\theta_{\boldsymbol{k}} - 2\sin\theta_\mathrm{B}\,\gamma(\theta_{\boldsymbol{k}},t) \end{pmatrix}. \end{aligned}\tag{76}$$

Equating this with

$$\hat{\boldsymbol{\kappa}}(\hat{\boldsymbol{k}},t) = R_{\boldsymbol{z}}(\varphi_{\boldsymbol{k}})\begin{pmatrix} \cos\Theta(\theta_{\boldsymbol{k}},t)\cos\Delta\Phi(\theta_{\boldsymbol{k}},t) \\ \cos\Theta(\theta_{\boldsymbol{k}},t)\sin\Delta\Phi(\theta_{\boldsymbol{k}},t) \\ \sin\Theta(\theta_{\boldsymbol{k}},t) \end{pmatrix}, \tag{77}$$

we read off the polar deflection function (58)

$$\Theta(\theta,t) = \arcsin\left(\sin\theta - 2\sin\theta_\mathrm{B}\gamma(\theta,t)\right), \tag{78}$$

and we define the azimuthal shift function

$$\Delta\Phi(\theta,t) := \operatorname{atan2}\left(-2\sin\theta_\mathrm{B}\,\beta(t),\ \cos\theta - 2\sin\theta_\mathrm{B}\,\alpha(\theta,t)\right). \tag{79}$$

The function $\operatorname{atan2}(y,x)$, as defined by the standard library of the programming language C, returns the principal value of $\arctan(y/x)$, using the signs of the two arguments to determine the quadrant. Thanks to the spherical coordinates, the rotation by $R_{\boldsymbol{z}}(\varphi_{\boldsymbol{k}})$ is trivial, so that we obtain the deflection function in spherical coordinates as

$$\theta_{\boldsymbol{\kappa}} = \Theta(\theta_{\boldsymbol{k}},t) \quad \text{and} \quad \varphi_{\boldsymbol{\kappa}} = \varphi_{\boldsymbol{k}} + \Delta\Phi(\theta_{\boldsymbol{k}},t). \tag{80}$$

B. Making matrix $\boldsymbol{L}$ quadratic

As discussed in Sect. 4.2, the linear equation system (38) is overdetermined. We therefore delete M equations out of a total of $M(N+1)$. The remaining equations are for $i=1,\ldots,N$, r forward, and for $i=0,\ldots,N-1$, r backward. The resulting index mapping is potentially confusing, and therefore merits accurate documentation.

To denote shifted indices in compact form, we define the indicator $\mathcal{F}r := [r \text{ is forward}] = [\hat{k}_{r,z} > 0]$. The collocation vector, after reduction from $(N+1)M$ to NM dimensions, shall be written $\tilde{p}_{ir} := p_{i+\mathcal{F}r,r}$ ($i=0,\ldots,N-1$), and similarly the matrix reduced from $((N+1)M)^2$ to $(NM)^2$, $\tilde{L}_{irjs} := L_{i+\mathcal{F}r,r,j+\mathcal{F}s,s}$. With this, (38) becomes

$$\forall_{i=0}^{N-1}\forall_{r=1}^{M}: \quad \sum_{j=0}^{N-1}\sum_{s=1}^{M}\tilde{L}_{irjs}\tilde{p}_{js} = \tilde{c}_{ir}, \tag{81}$$

which are NM equations in NM variables. On the right-hand side,

$$\tilde{c}_{ir} := -\sum_{s}^{\text{forward}} L_{i+1,r,0,s}J_{\mathrm{in},s} \tag{82}$$

can be evaluated as

$$\tilde{c}_{ir} = \begin{cases} -2D_{i+1,0}\hat{k}_{r,z}J_{\mathrm{in},r} & \text{if } r \text{ is forward,} \\ \delta_{i0}\sum_{s}^{\text{forward}} dB_{rs}J_{\mathrm{in},s} & \text{if } r \text{ is backward.} \end{cases} \tag{83}$$

The matrix $\tilde{L}$ has the entries

$$\tilde{L}_{irjs} = \begin{cases} 2\hat{k}_{r,z}D_{i+\mathcal{F}r,j+\mathcal{F}r} + dA_r\delta_{ij} & \text{if } r=s, \\ -dB_{rs}\delta_{i+\mathcal{F}r,j+\mathcal{F}s} & \text{else.} \end{cases} \tag{84}$$

C. Sequential allocation

For representing $\tilde{L}$ in sparse-matrix form, and for solving the equation system (81), we rely on Trilinos. Trilinos, as any other library, requires input and output

vectors to have standard one-dimensional array form. Therefore we need to combine the collocation index $i = 1, \ldots, N$ and the directional index $r = 1, \ldots, M$ into one single storage location index [6, ch. 2.2.6]. This can be done in different ways, for instance

$$\mathrm{loc}(i, r) \coloneqq iM + r. \tag{85}$$

Our directional grid has two disjoint domains, around the mean transmitted and deflected beam. Both subgrids are related by mirror or inversion symmetry, and have the same size $M/2$. Accordingly the grid is indexed by a pair $r = (r', r'')$, where $r' = 0, 1$ designates the subgrid, and $r'' = 0, \ldots, M/2$ is the index within a subgrid. Among all six permutations of r', r'' and i, the mapping

$$\mathrm{loc}(i, r) \coloneqq r' \frac{MN}{2} + i \frac{M}{2} + r'' \tag{86}$$

turned out to make the preconditioner fastest.

---

[1] F. Bornemann, Y. Y. Li and J. Wuttke, Acta Cryst. A **in press** (2020).

[2] D. van Heesch, *Doxygen: Generate documentation from source code,* `http://www.doxygen.org` (1997–2018).

[3] M. Heroux, R. Bartlett, V. Howle, R. Hoekstra, J. Hu, T. Kolda, R. Lehoucq, K. Long, R. Pawlowski, E. Phipps, A. Salinger, H. Thornquist, R. Tuminaro, J. Willenbring and A. Williams, *An Overview of Trilinos. Report SAND2003-2927*, Sandia National Laboratories: Albuquerque (2003).

[4] J. Wuttke, *`Frida`: Fast reliable interactive data analysis,* `https://jugit.fz-juelich.de/mlz/frida`.

[5] J. Wuttke, Acta Cryst. A **70**, 429 (2014).

[6] D. E. Knuth, *The Art of Computer Programming. Vol. 1: Fundamental Algorithms*, Addison-Wesley: Upper Saddle River (^{3}1997).
